# Health and medical apps - Same same but different? A review of definitions in public health and law

**DOI:** 10.1093/eurpub/ckac131.165

**Published:** 2022-10-25

**Authors:** L Maass, M Freye, C-C Pan, H-H Dassow, J Niess, T Jahnel

**Affiliations:** 1 Leibniz ScienceCampus Digital Public Health, Bremen, Germany; 2 Research Center on Inequality and Social Policy, University of Bremen, Bremen, Germany; 3 Institute for Information, Health and Medical Law, University of Bremen, Bremen, Germany; 4 Epidemiological Methods & Etiological Research, University of Bremen, Bremen, Germany; 5 Institute for Philosophy, University of Bremen, Bremen, Germany; 6 Human-Computer-Interaction, University of St. Gallen, St. Gallen, Switzerland; 7 Institute for Public Health and Nursing, University of Bremen, Bremen, Germany

## Abstract

The terms health app and medical app are often used interchangeably but do not necessarily mean the same. Medical apps can be regulated as mobile medical devices and therefore need to meet general safety and performance requirements. On the contrary, health apps are not part of this legal framework and do not need proof of their efficacy or guaranteeing data security. We need distinct definitions of health and medical apps to understand these terms better and regulate such technologies more effectively. We will provide an overview of health and medical apps definitions and a differentiation flowchart from public health and legal perspectives. A search in 6 databases identified 22 publications that defined health apps and 11 reports that described medical apps. The core elements of these definitions were identified through qualitative analysis. Health and medical apps share the same devices, technical functions and collect health data. While it can be highly challenging to decide which legal requirements have to be fulfilled by an app, we deem it unfit as a distinction criterion. It requires legal knowledge, which is neither suitable nor practical for users. However, medical device law is closely linked to the app’s intended medical or health purpose criteria, which allows a clear differentiation. Additionally, the difference in the user group can be used for separation. Our suggestion for the definition would be that health apps are software programs on mobile devices that process health-related data on/for their user. They can be used by every health-conscious person to maintain, improve or manage the health of an individual or the community. As an umbrella term, health apps include medical apps. They share the same technological functions and devices as health apps. Health professionals, patients, and family caregivers are the main user groups of medical apps. Due to the intended use for clinical purposes, medical apps can be regulated as mobile medical devices.

**Key messages:**

• Separating apps based on legal regulation is impractical & can’t be expected from all stakeholders. Differencing between health and medical apps requires the user group and the health aim of the app.

• Health apps include medical apps. Since 2019, medical apps in Germany contain digital health applications (DiGA). Since 2022, medical apps partly include digital care applications (DiPA).

